# Bovine Tuberculosis (*Mycobacterium bovis*) Outbreak Duration in Cattle Herds in Ireland: A Retrospective Observational Study

**DOI:** 10.3390/pathogens9100815

**Published:** 2020-10-05

**Authors:** Andrew W. Byrne, Damien Barrett, Philip Breslin, Jamie M. Madden, James O’Keeffe, Eoin Ryan

**Affiliations:** 1 One-Health Scientific Support Unit, Surveillance, Animal by-products, and TSEs (SAT) Division, Department of Agriculture, Food and the Marine, Agriculture House, Dublin 2 D02 WK12, Ireland; damien.barrett@agriculture.gov.ie; 2 Ruminant Animal Health Division, Department of Agriculture, Food and the Marine, Backweston, Co. Kildare W23 VW2C, Ireland; philip.breslin@agriculture.gov.ie (P.B.); james.okeeffe@agriculture.gov.ie (J.O.); 3 Centre for Veterinary Epidemiology and Risk Analysis (CVERA), School of Veterinary Medicine, University College Dublin, Belfield, Dublin 4 D04 W6F6, Ireland; jamie.madden@ucd.ie

**Keywords:** bovine TB, risk factors, disease control, animal health policy, veterinary epidemiology, evidence-based policy

## Abstract

Bovine tuberculosis (bTB) outbreaks, caused by *Mycobacterium bovis* infection, are a costly animal health challenge. Understanding factors associated with the duration of outbreaks, known as breakdowns, could lead to better disease management policy development. We undertook a retrospective observational study (2012–2018) and employed Finite Mixture Models (FMM) to model the outcome parameter, and to investigate how factors were associated with duration for differing subpopulations identified. In addition to traditional risk factors (e.g., herd size, bTB history), we also explored farm geographic area, parcels/farm fragmentation, metrics of intensity via nitrogen loading, and whether herds were designated controlled beef finishing units (CBFU) as potential risk factors for increased duration. The final model fitted log-normal distributions, with two latent classes (k) which partitioned the population into a subpopulation around the central tendency of the distribution, and a second around the tails of the distribution. The latter subpopulation included longer breakdowns of policy interest. Increasing duration was positively associated with recent (<3 years) TB history and the number of reactors disclosed, (log) herd size, beef herd-type relative to other herd types, number of land parcels, area, being designated a CBFU (“feedlot”) and having high annual inward cattle movements within the “tails” subpopulation. Breakdown length was negatively associated with the year of commencement of breakdown (i.e., a decreasing trend) and non-significantly with the organic nitrogen produced on the farm (N kg/hectare), a measure of stocking density. The latter finding may be due to confounding effects with herd size and area. Most variables contributed only moderately to explaining variation in breakdown duration, that is, they had moderate size effects on duration. Herd-size and CBFU had greater effect sizes on the outcome. The findings contribute to evidence-based policy formation in Ireland.

## 1. Introduction

Bovine tuberculosis remains a priority pathogen of cattle in Ireland [1,2] and elsewhere [3,4,5,6] and one that expends considerable resources from farmers, the state, and, for member states, the EU Commission. As an example, direct annual programme costs in Ireland were estimated at €92 million in 2018, €47 million from the Exchequer, €9.7 million co-financed by the EU, and €35.2 million paid by farmers [7]. A key challenge is to ensure that disease policy is evidence-based and adapts in response to changes in risk patterns so that programme effectiveness is maximised and stakeholders can be assured that decisions affecting them are underpinned by a coherent epidemiological analysis of the data [8].

The duration of breakdowns can have a significant financial impact on stakeholders [9,10], and furthermore, prolonged duration breakdowns can be a risk factor for future recrudesce [2]. Therefore, understanding risk factors associated with prolonged duration can be of benefit to tailoring policy to local conditions, with the intentions of reducing the spread of infection within and between herds.

In Ireland, a significant expansion of the dairy sector took place from 2015 onwards following the removal of EU milk quotas, with an increase of 54% in milk production from 2005 to 2018 [11]; this has been temporally correlated with an increase in TB herd incidence. The increase in dairy production has been associated with an increase in stocking density and herd size, while studies characterised the scale of cattle movements within Ireland [12], it is unclear whether the nature of how these factors relate to TB risk has also changed in that period [13]. However, data from Britain suggest such movement increases may increase inter-herd *M. bovis* spread [14].

Duration data are positive integers (natural numbers) data with often unusual distributions which can be difficult to model using conventional statistical means. Previous attempts to model breakdown duration has used categorical models where duration is split into binary outcomes [15] or ordinal outcomes [10]. Breakdown duration has also been modelled as a count of days, using a negative binomial random-effects model [10]. Time to event modelling (survival) models can be used to model the interval between failures [15,16]. Alternatively, the duration of time over a fixed period can be modelled by generalised linear models, using various distribution types (e.g., hospitalisation length of stay [17]). Breakdown duration that is the period over which herds are not able to trade due to the presence of TB infection within the herd, in Ireland is both leptokurtic and positively skewed. To explore the variation in breakdown length, a two-class Finite Mixture Model (FMM) was fitted to the data. FMMs are probabilistic models that partition populations into unobserved subpopulations, known as classes or components, without explicitly identifying the subpopulation for which observations belong. Instead, subpopulation membership is modelled as a latent class, from which the posterior class probability of membership can be estimated, and mixtures of continuous probability distributions of various types (regression models) are used to model the outcome allowing for statistical inference at the subpopulation level. Therefore, the FMM is a flexible approach to modelling heterogeneous data [18].

The study also aimed to investigate different characteristics of herds that may impact on their ability to clear the infection, and thus impact breakdown duration so that the controls may be tailored to the risk of persistence of infection. We investigated parameters that have been found to be associated with breakdown duration in other countries, including previous breakdown history, herd type, and herd size, but also explored additional potential predictors. Herd size (i.e., the number of animals on the farm) has been consistently found to be associated with TB breakdown risk, recurrence and duration [9,10,19,20,21,22,23,24]. However, herd size could be a proxy for several other farm characteristics [25], for example, the density of animals (e.g., animals per hectare) and the intensity of farming (e.g., high input-high output farms) which could be associated with TB transmission dynamics and maintenance. The geographic area of the farm and configuration (e.g., number of land parcels) could also impact on farm risk of exposure to *M. bovis* from contiguous spread from neighbours or wildlife sources [23]. Therefore, we have added additional proxies for these factors as potential risk factors in our model. This work, therefore, also helps to tease out what aspects of herd size that are associated with breakdown duration in Ireland. Controlled beef finishing units (CBFU), colloquially known as “feedlot” herds, were also identified and modelled as a factor influencing breakdown duration. Previous research elsewhere has shown how such farming systems can be associated with increase prolongation of breakdown duration, associated with increasing genotype diversity of *M. bovis* facilitated through trade movements [10,26]. Animals from such herds go straight to slaughter, and therefore, are limited in terms of onward interherd transmission but are important to identify to explain variation in our outcome of interest. Overall, this study contributes towards informing evidence-based changes to bTB policy to address the risks associated with changing herd dynamics.

## 2. Results

**Figure 1:**
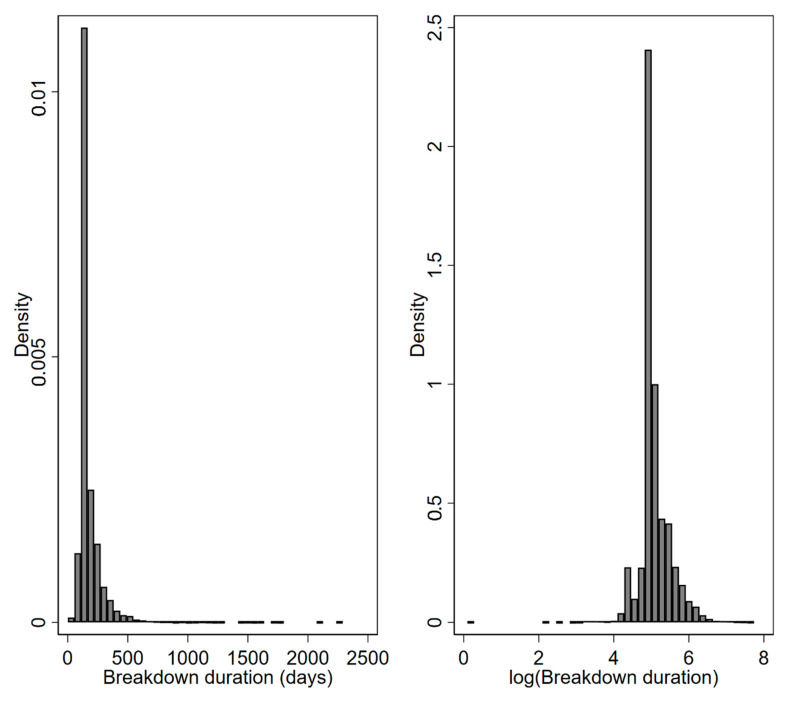
Breakdown duration days (**left**) and with duration log-transformed (**right**).

The final dataset included 17,312 breakdowns. The mean breakdown length was 177.85 days (SD: 105.25; Median: 147; IQR: 135–187; Figure 1). The distribution of breakdown durations was both leptokurtic (kurtosis: 68.24) and positively skewed (skewness: 5.90; Figure 1).

### 2.1. Model Distribution and Class Probability

**Figure 2:**
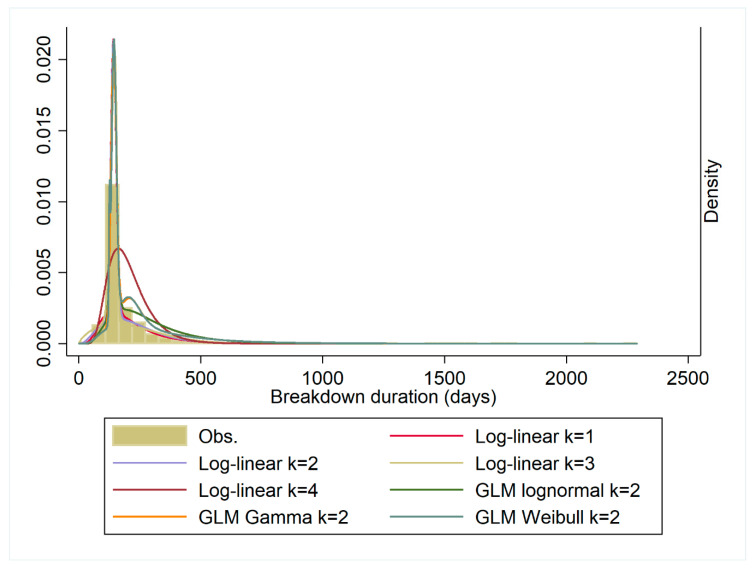
A comparison of Finite Fixture Models (FMMs) with differing distributions to model classes (OLS log-linear k = 1; OLS log-linear k = 2; OLS log-linear k = 3; OLS log-linear k = 4, GLM lognormal k = 2; GLM gamma distribution model k = 2; GLM Weibull distribution model k = 2).

Distribution fits to null models (that is, without covariates) were compared using BIC and AIC, with the following FMM models compared (Figure 2): 1. Ordinary Least Squares (OLS) log-linear; 2. GLM lognormal; 3. Gamma distribution; 4. Weibull distribution (Table 1). GLM Poisson and Negative binomial models did not converge. OLS models, where the outcome was natural logarithm transformed, were best-fitting models with the smallest AIC and BIC. There was evidence also that FMM models (with k > 1) were favoured in comparison with a model without latent subpopulation classes.

Comparing the number of classes (k) to fit the data, a three-class and four-class model fitted the data better, with a lower BIC and AIC relative to the two-class model (Table 1). However, the parsimony of the two-class models was preferred. Furthermore, research has shown the overfitting may be an issue for mixture models where k > 2, and convergence for more complicated models can be an issue for mixture models. The best-fitting model with k = 2 was the log-linear regression model [outcome variable was log-transformed before fit using the REGRESS command].

**Figure 3:**
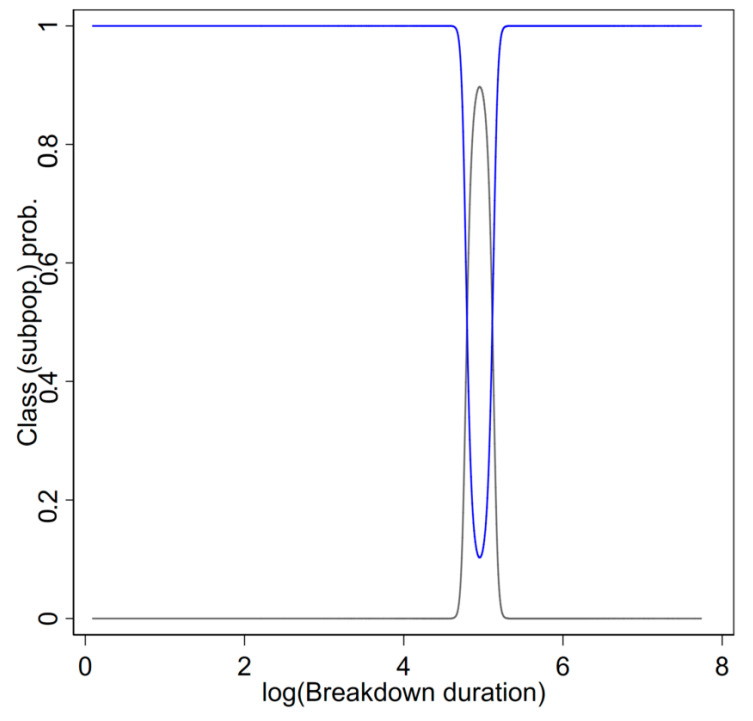
The predicted probability of class membership for a two-latent class FMM null model fitted to bovine tuberculosis (TB) breakdown duration data. Grey line represents class 1; blue line represents class 2.

**Figure 4:**
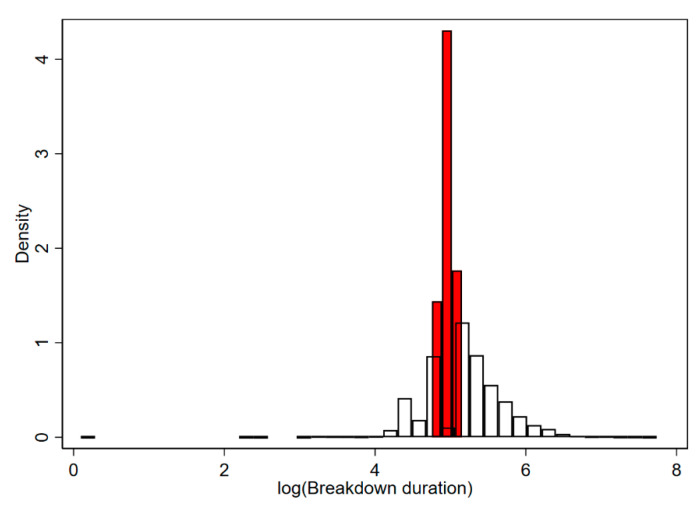
The predicted density from an FMM with two classes fitted to bovine TB breakdown duration data; class 1 is represented as red bars which cluster around the central tendency of the distribution; class 2 is represented as clear bars in the histogram which encapsulated more of the variance in the dataset away from the mean.

The two latent distributions fitted to different parts of data distribution (Figure 3). Figure 3 presents the predicted posterior probability of class membership for class 1 (black line) and class 2 (blue). The probability of class 1 membership (relative to class 2 membership) was very high for very large and small values. Class 2 membership was limited primarily to a narrow range around the central tendency. These data are visualised in Figure 4 by plotting the histograms of data with > 20% probability of membership for PR2 class (clear) and PR1 class (red) (Figure 4).

### 2.2. Development of a Multivariable Log-Normal FMM

A baseline model was developed with a fixed set of covariates log-herd size, herd-type, and herd history (breakdown history over the previous 3 years), as potential predictors of log-breakdown length based on previous research. The probability of being class/component 1 or 2 was modelled as a function of log-herd size, herd history and herd type, as we hypothesized that breakdown duration may be associated with these factors. Comparisons were made using BIC and AIC with baseline models with the inclusion or exclusion of these factors, separately and respectively. Baseline models had the lowest BIC values when all three factors were retained to explain variation in breakdown length, and when both log-herd size and herd type were retained as factors explaining some variation in the probability of being associated with class 1 or 2 (Baseline vs. class prob (log_hs, herd_type) ΔBIC: 43.75; Table 2). The model with the lowest AIC included herd-history as an additional predictor of class probability, but the more parsimonious (BIC) model was selected.

The final model outputs are presented in Table 3 and Table 4. Predictions from the model (Figure S1) showed that class 1 were tightly bound around the central tendency of the distribution, “central” subpopulation; class 2 had greater representation at the tails of the distribution, “tail” subpopulation. The entropy value, which discerns how distinct subpopulation classes were, was estimated at 0.58, which is moderate but reasonable for a large population size with 2-classes. The subpopulations were reasonably balanced, with 51% of the population classified as Class 1 “central” subpopulation, and 49% being falling into the Class 2 “tail” subpopulation. The (geometric) mean breakdown duration for Class 1 subpopulation was 142 days (exp(4.96)), while for subpopulation Class 2 the mean duration was 183 days (exp(5.21)).

The preferred model with the lowest BIC retained both log-herd size and herd type as explaining variation in the probability of herds being associated with either latent class component. Table 3 shows the output of this part of the mixture model. The model suggested that increasing log-herd size was significantly associated with having a greater probability of being in Class 2 “tail” subpopulation. Dairy herds had a significantly greater probability of being represented in Class 2 “tail” component, relative to Class 1 “central”, in comparison with beef rearing/finishing production type (*p* = 0.010). “Other” herd types had a lower probability of being in class 2 relative to beef herd types (*p* < 0.001). There was no significant difference in class probabilities between beef rearing/finishing and beef suckler (*p* = 0.061) herd type.

The associations between factors hypothesized to impact breakdown duration for Class 1 and Class 2 are presented in Table 4. All predictors were retained in the log-normal regression parts of the model explaining breakdown duration for both components/class 1 and 2. Comparing coefficients between Class 1 and Class 2 suggests that risk factors may have a different associated effect on breakdown duration.

Class 1 breakdowns duration tend to decrease with increasing nitrogen load per hectare (log; log_NPH) and for breakdowns commencing later in the time series (bd_start_yr). The duration was positively associated with herd history (herd_history), with herds with breakdowns during the previous three years having a longer duration than those without breakdowns. There was not, however, a clear dose-dependency in terms of breakdown size (i.e., number of reactors disclosed). Herds with 1, 2–4, or > 4 reactors were a significantly higher risk for longer breakdowns within this “central” subpopulation, relative to herds without reactors. There was no difference in breakdown duration between herds without a previous breakdown and “zero-reactor” breakdowns (i.e., historic breakdowns that were disclosed due to a TB lesion detected at routine slaughter and in which no additional reactors were disclosed at follow-up herd tests). There was no significant association between duration and herd size, CBFU (feedlot) status or farm fragmentation (parcel_cut). Inward movements were dichotomised into herds which had high mean yearly inward movements (>90 moves) or lower mean inward movements (<90). In Class 1 subpopulation, there was a negative association between high inward movements and breakdown length (−0.039; 95% CI: −0.04–−0.032; 3.8% reduction in duration).

**Figure 5:**
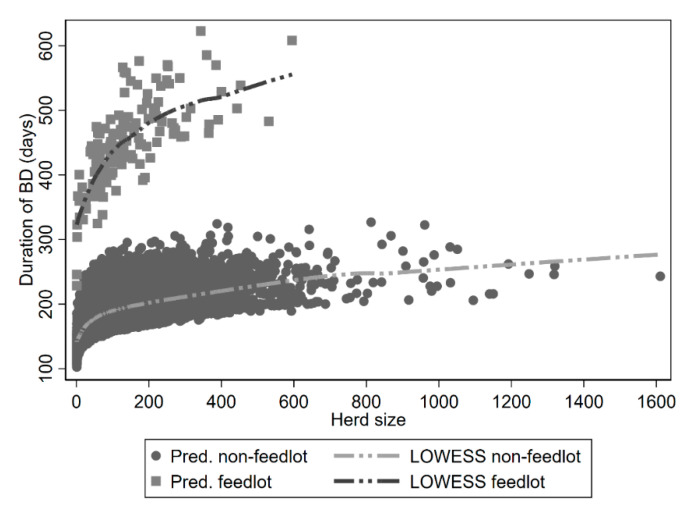
Relationship between herd size and breakdown duration as predicted for Class 2 subpopulation of a finite mixture model. Squares are predictions for herds with a controlled beef finishing unit “feedlot” designation; Points are predictions for herds without “feedlot” designation. Lines are Local Weighted Scatterplot Smoothing (LOWESS) curves fitted to the data.

Herds associated with class 2 “tail” subpopulation exhibited significantly increased breakdown duration with increasing herd-size (log_hs), and if designated with a “feedlot” status (CBFU). Duration of breakdowns increased by 9.4% (β: 0.090; 95% CI: 0.077–0.102) for every log-unit increase in herd size. Feedlot herds within class 2 subpopulation were 203.4% (β: 0.710; 95% CI: 0.603–0.818) longer duration relative to non-feedlot herds. The median predicted duration for feedlot herds was 451 days, whereas as the median duration was 182 days for non-feedlot designated herds. The predicted relationship for this subpopulation for herd size and feedlot designation is presented in Figure 5.

**Figure 6:**
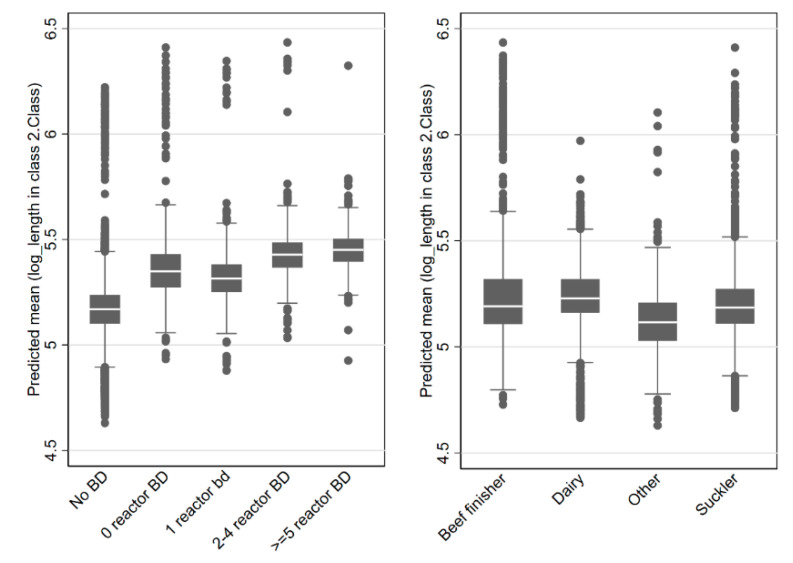
Relationship between breakdown history during the previous 3 years and predicted mean breakdown duration (top). Relationship between herd type and predicted mean breakdown duration (bottom). Note, log scale.

Duration increased if a herd had previously experienced a breakdown during the previous 3-years. There was a trend towards dose-dependency, with increasing duration associated with a greater number of reactors disclosed during the previous breakdown (Figure 6), however, the size effect was reasonably small. For example, there was a 25% (β: 0.226; *p* < 0.001; 95% CI: 0.155–0.296) increase in mean duration for the herd with five or more reactors relative to herds which did not experience a breakdown during the previous 3 years.

Parcel number (parcel_cut), a measure of farm fragmentation, was positively associated with increased breakdown length, though only for herds with 4 or more parcels relative to herds with 1. Again, the effect size was moderate, for example, a 6.6% (β: 0.064; *p* < 0.001; 95% CI: 0.023–0.105) increasing duration for herds with 4 parcels relative to herds with only 1 parcel.

Beef rearing/finishing herds had significantly longer breakdown duration relative to dairy, suckler or “other” herd types (herd_type) (Table 4; Figure 6). For example, dairy herds experienced mean breakdown log-lengths the were 7.2% shorter (exp(β: −0.138)-1) relative to beef herds.

The linear predictor for start year of breakdown (bd_start_yr) suggested that breakdown lengths decreased slightly per year of the study for Class 2 subpopulation (0.5% reduction per year; β: −0.023; *p* < 0.001; 95% CI: −0.017–−0.029).

Controlling for herd size, intensity metric based on nitrogen loading (NPH) was negatively associated with breakdown length, though this effect was not significant (β: −0.014; *p* = 0.054; 95% CI: −0.028 < 0.001). It was likely that the effect was impacted by confounding with herd size and area.

In Class 2 subpopulation, there was a positive association between breakdown length and high inward movement (0.108; 0.062–0.155; 11.4% increase in duration). The size effects for both subpopulations are reasonably small, for example, herds in subpopulation 2 increase their mean duration by approximately 20.8 days if they make > 90 inward moves per annum (i.e., mean [183 days] × 0.114).

## 3. Discussion

This study was the first to explore variation in breakdown duration explicitly in Ireland, and more generally the first to use finite mixture models (FMM) to explore the variation in breakdown duration for bTB. It is also the first study to investigate cattle production of nitrogen per hectare (a common measure of the intensity of livestock production within the EU) as a potential factor associated with bovine TB breakdown duration. The significance of this approach lies in enabling the adaption of disease policies to change risks based on an evidence base which is inclusive of larger trends such as livestock intensification.

Previous research has dichotomised breakdown duration and modelled using logit distributions (e.g., [9,15,26,27]), have broken down duration into ordered categories of increasing duration and modelled using ordinal regression models [10] or have modelled duration as a count of days using a negative binomial discrete distribution [10] or using simulation approaches [28]. The durations recorded in the present study exhibited an unusual distribution, with both positive skewness and kurtosis. The mixture model allowed for two different distributions to be fit to the data, with class associations being modelled via a latent class model.

The latent classification of the data into two classes suggested that observations were clustering tightly around the central tendency and a second which captured more of the variation in the outcome, especially at the tails of the distribution. The latent component of the model suggested that larger herds and dairy, relative to beef, herds had a higher probability to be represented by the tail subpopulation, relative to the central subpopulation. This may indicate that such herd characteristics are associated with more unusual breakdowns away from the central tendency. Previous research has found that chronic breakdowns, which would be represented by our tails subpopulation, can be associated with both increasing numbers of animals within the herd (e.g., [9]) and dairy enterprises ([27,29]) relative to other herd types.

In terms of interpretation, the latent classification and the “tail” subpopulation log-normal regression model are informative. The log-normal regression for the ‘central’ subpopulation is less informative, from a policy perspective, as 1. It seeks to explain variation over a narrow range; that is, the variance in the outcome was low, 2. Larger breakdowns are of greater significance from a disease policy development perspective, assuming that at least partially the speed at which average breakdowns clear may be more related to breakdown management/compliance and response times (e.g., actions by the farmer and operational delivery by veterinarians) as well as epidemiologically relevant factors. However, it is interesting to note the differences in the relationship between the outcome and predictors between subpopulations. In the central subpopulation, there was no significant association with duration and herd-size, number of land parcels, controlled finishing units (feedlots) or breakdown starting year. Dairy herds varied in their association with breakdown duration across subpopulations, with longer durations relative to beef herds in subpopulation 1, and shorter durations relative to beef herds in subpopulation 2. However, it should be borne in mind that dairy herds have a higher class-probability for subpopulation two. Furthermore, dairy herds tend to be larger and have older age profile animals, both of which are associated with bTB risk [22,28].

The class 2 log-normal part of the FMM suggested that breakdown lengths were significantly increased with increasing numbers of reactors disclosed at the breakdown during the previous 3 years. Herd history has been associated with breakdown length in previous studies outside of Ireland, though metrics of TB history were not always analogous to what was done in the present study [10,15]. A history of TB in a herd was associated with large (>13 reactors) bTB breakdowns, relative to small (2–4 reactors) in a case-control study in Ireland [30]. Interestingly, recent work suggested that that previous breakdown size was not associated with recurrence risk in Ireland [2].

The factors that appeared to have the largest effects on breakdown duration were herd-size and whether the herd was ever designated a controlled beef finishing unit “feedlot” herd. Controlled finishing unit herds are non-breeding beef finishing units which are only permitted to move cattle directly to slaughter and fulfil certain criteria to receive the designation, for example, biosecurity protocols are in place, and boundaries are double fenced or equivalent to prevent any direct contact with cattle on neighbouring lands/premises/holdings. Such herds may apply for controlled finishing unit herd designation upon breakdown, subject to there being no evidence of within-herd spread; breakdowns are typically due to lesions detected at routine slaughter from cattle bought-in and fattened over a relatively short period of time for finishing. This status enables them to continue to buy in beef cattle for finishing during breakdowns. These herds are deemed of lower risk for onward infection transmission due to high turnover of animals within the herds before going direct to slaughter. The effectiveness of controls to prevent such herd enterprises posing a risk to neighbouring herds via wildlife spillover or contiguous spread is subject to another ongoing study (J. Madden pers. comm.). Beef fattening herds in Northern Ireland, which can buy-in animals during breakdowns, have increased strain diversity of *M. bovis* and increased breakdown duration time, linked to inward animal movements from a wide geographical area [10,31]. The association between such herds and increased breakdown length identified in this study and in Northern Ireland may be explained by the ability of the business model to continue while under restriction; the length of breakdown does not reflect a chronic disease problem on these farms per se but rather the reality that a farm practice which involves purchasing in large numbers of beef cattle for fattening and slaughter makes it likely that the detection of bTB lesions at routine slaughter would be a regular occurrence, given the background level of bTB in Ireland. Each such detection extends the breakdown again, lengthening breakdown duration but not necessarily reflecting the continuing spread of infection within the herd.

Herd size has been found to be a significant factor for TB breakdown risk in general for many studies (e.g., see [3,23]). Brooks-Pollock and Keeling [22] showed how herd size was associated with breakdown persistence, which the authors interpreted as indicating a lack of clearance of infection within herds after a breakdown. We have attempted to explore what correlates and related variables of herd size that might be important for breakdown duration with the inclusion of nitrogen load (a measure of agricultural intensity, based on organic nitrogen per hectare), land area, and parcel number. For Class 2, “tail” subpopulation, there were significant increases in log-length of breakdown with increasing land area and the number of parcels (primarily 4 or more parcels, relative to single parcel units), while controlling for herd size. However, for both factors, the effect size was small. Despite this, previous work elsewhere has found significant positive associations between farm fragmentation [32,33,34] and land area [33,35] and TB breakdown risk, though there are little comparative data on these factors relationship with breakdown duration.

Surprisingly, controlling for herd size there was a negative relationship with increasing nitrogen load. This outcome is most likely affected by the significant confounding relationship between herd size, area and nitrogen load (see Figure S2). Indeed, nitrogen per hectare (NPH) was positively and significantly associated with breakdown duration at the univariable level but not significantly in a multivariable model without area or herd size (Figure S3; Table S1). The final multivariable model suggested that herd size, that is the number of animals/potential hosts, appears to be the key herd-level factor for predicting breakdown length.

It was encouraging that for both subpopulations that the temporal trend was for a reduction in mean duration over time. This suggests that interventions aimed at clearing infection once reactors are identified are being more effective over time. However, it should be noted that the estimated reduction is very small, being less than a day reduction in mean duration for the class 2 subpopulation. Previous work in Ireland has found a decreasing trend also in breakdown recurrence from 1998–2015 [36], though recent trends suggest the incidence is increasing.

In conclusion, our work has suggested breakdown duration in Ireland is related to several herd and farm characteristics, including the herd size, type, area (acreage), fragmentation, and trading frequency. Given that herd size and acreage contributed additively to increasing breakdown duration, trends in farming including farm expansion and consolidation may signal future increases in breakdown duration, causing a greater challenge to the bTB control program.

## 4. Materials and Methods

### 4.1. Data

The dataset used is a breakdown herd dataset where each line in the dataset represents a discrete breakdown [37]. Throughout, the start date and end date within that file was used to define breakdown durations (see [37] for additional detail). All breakdowns were initiated by the disclosure of (i) bTB reactors from Single Intradermal Comparative Cervical Tuberculin test (SICCTT) or (ii) the disclosure of a bTB lesion found at slaughter (factory lesion). End dates were when herds were derestricted, in compliance with the EU regulations on bovine TB. The dataset included breakdowns that were ongoing between January 2012 and December 2018 and were initiated from January 2012–December 2018. Follow-up period ended in August 2019.

Two inclusion criteria were employed to the data. Only the most recent breakdowns per herd were selected; this was to avoid repeated measures per herd and therefore simplify the analysis. All breakdowns that were censored by the follow-up (i.e., they were still experiencing a breakdown when follow-up finished in August 2019) were removed from the dataset.

All data are available upon reasonable request from corresponding authors.

### 4.2. Model

Finite Mixture Models (FMM) are a flexible modelling framework that have been applied to several biological problems, including clustering, estimation of diagnostics performance, and for modelling heterogeneous data [18]. In addition, FMM is an approach that can be applied to duration models [17,18,38]. Applications of an FMM model to duration include modelling length of stay in hospitals [17] and age-at-onset for various conditions in human medicine including migraine or bipolar disorder (e.g., [39,40,41]). FMMs give flexibility to fit the data by allowing admixture of distributions, called classes (k) to explain the variation in the data. Furthermore, different continuous probability distribution types can be fitted, including normal, log-normal, Poisson, negative binomial, Gamma and Weibull. For example, log-normal distributions have been applied to “survival” type data, as an alternative to Cox proportional hazard models [42]. Here we developed a model of breakdown duration without repeated measures using an FMM approach.

Models were compared using Bayesian information criteria (BIC) and Akaike’s Information Criteria (AIC) values, which favour models which explain variation in our outcome while penalising models with many vaguely informative parameters. Where BIC and AIC did not support the same model configuration, the model with the least parameters were preferred, which was generally the BIC preferred model. Simulation studies have shown that BIC is generally preferred to AIC for FMM, as AIC can tend to select for too many classes [43].

Entropy value, which discerns how distinct subpopulation [latent] classes were, was estimated [44]. The higher the number the greater the separations between classes, “rule of thumb” threshold of 0.8 has been proposed. However, recent simulation analysis has shown that entropy values are sensitive to sample size. Entropy values < 0.2 might be expected in large sample studies (e.g., >3000 observations, 1500 per subpopulation) with balanced spilt between 2-classes and where true sample separation has been simulated [44].

Our process was to plot out the distribution of breakdown durations and fit differing FMM types, including log-normal, normal, and count distributions. We explored k > 2 class models, but only retained a two-class model for multivariable models to ensure a more parsimonious model that was more meaningful from an epidemiological perspective. Furthermore, a higher number of components in FMMs are associated with overfitting [45].

The steps involved in the construction of the model is depicted in Figure S4. Once we developed a well-fitting null model without covariates, we built multivariable FMMs. Initially, baseline models were fit with the parameters: herd size, herd history, and herd types. Then additional parameters were added sequentially. Comparisons between model configurations were made using AIC/BIC values; lower values indicated better performing models. Outcome and covariate parameter summary statistics are presented in Table 5.

Additional predictors of breakdown duration were added to this model. The model fitted a controlled beef finishing unit proxy “feedlot” status (binary; 1 if herd ever had designation as a controlled beef finishing unit; 0 otherwise), an intensity metric based on nitrogen loading per hectare (logarithm of median bovine nitrogen per hectare over 2015–2018), the number of parcels (recorded per herd in 2018 using LPIS at a resolution of 7 m), land area (median hectares over 2015–2018), and breakdown start year, and inward animal movement metric. The mean number of inward animal moves (2013–2018) into a farm was dichotomised into “large” (mean ≥90 per annum) and other (<90 mean moves).

Herd size was log-transformed. Herd history was a categorical variable indicating whether the herd had previously had a breakdown within 3-years, and if so, the size of the breakdown in terms of reactors disclosed (0, 1, 2–4, ≥5). Breakdowns with 0 reactors would have been initiated by lesions found during slaughter at abattoir during post-mortem surveillance [37]. Herd types were taken from the AHCS dataset, as beef, dairy, “other” or sucker herds. Herd type was based on standard definitions used by DAFM: dairy herds are self-explanatory; beef herds are non-breeding herds; suckler herds are beef breeding herds; the designation “other” reflects herds which engage in a combination of such activities.

Other variables offered to the models included: controlled finishing unit (colloquially referred to in Ireland as “feedlots”) status, nitrogen loading, number of farm fragments/parcels, number of moves into the herd, and year of breakdown initiation.

“Feedlot” status was assigned if a herd was ever designated a controlled beef finishing unit (CBFU) by DAFM at the breakdown from 2006–2018 (see Discussion).

Nitrogen, measured as the organic nitrogen loading per hectare (NPH) from bovines, was measured as the median NPH per herd over 2015–2018 assigned to each herd. Because of the skew of this variable (a small number of herds with very large NPH values) the parameter was log-transformed. These data were estimated by DAFM in accordance with Nitrogen Regulations in Ireland.

The number of farm parcels was measured from the Land Parcel Identification System (LPIS) 2018, with discrete land-parcels being classified if parcels were >7 m from each other. This buffer was chosen to ensure that parcels that effectively were single units were not counted as separate parcels. Land parcels were categorised into 1, 2, 3, 4 or ≥5 parcels.

Land area was measured as the mean hectares land area declared during 2015–2018 for Basic Payment Scheme (BPS) purposes. The median of these values per herd was assigned as a metric of geographic footprint for each herd in the dataset. There was a 99% correlation between individual yearly and median land area.

Movement of animals into herds were measured by a single metric in this instance, as the mean of total inward moves per annum from 2013–2018, inclusive. Univariable models suggested that most variation in the relationship between breakdown length and inward moves was whether herds brought in large numbers of cattle or not. Therefore, the data were categorised into herds with an average of > 90 inward moves per year and those with <90 inward moves per year.

Year of breakdown initiation was entered into models initially as categorical variables but added many parameters (n = 7 parameters; 2012–2018). The exploratory analysis suggested that trends could be modelled as a single linear predictor.

All analysis was undertaken in Stata 15.1 MP–Parallel Edition (Stata Corp., 2017) using the FMM suite of commands.

## Figures and Tables

**Table 1:** Comparison of Bayesian and Akaike’s Information Criterion values for finite mixture models fitted to bovine TB breakdown duration data. k = number of latent classes (subpopulations).

Model	BIC	AIC
**Log(duration), k = 1**	17,678.83	17,663.32
**Log(duration), k = 2**	6532.47	6493.67
**Log(duration), k = 3**	6092.97	6030.90
**Log(duration), k = 4**	5737.85	5652.50
**GLM lognormal, k = 2**	182,664.06	182,625.26
**GLM gamma, k = 2**	183,161.31	183,122.52
**GLM Weibull, k = 2**	185,397.98	185,359.19

**Table 2:** Comparison of base FMM models using Bayesian and Akaike’s Information Criteria.

Model	Class Probability	Regression Predictors	BIC	AIC
**Log(duration), k = 2**	.	log_hs herd_history herd_type	5893.03	5730.09
**Log(duration), k = 2**	log_hs	log_hs herd_history herd_type	5858.36	5687.66
**Log(duration), k = 2**	herd_type	log_hs herd_history herd_type	5853.46	5667.24
**Log(duration), k = 2**	herd_history	log_hs herd_history herd_type	5924.37	5730.39
**Log(duration), k = 2**	log_hs, herd_type	log_hs herd_history herd_type	5849.29	5655.31
**Log(duration), k = 2**	log_hs, herd_history	log_hs herd_history herd_type	5884.57	5682.83
**Log(duration), k = 2**	herd_type, herd_history	log_hs herd_history herd_type	5879.33	5662.08
**Log(duration), k = 2**	log_hs, herd_type, herd_history	log_hs herd_history herd_type	5872.54	5647.52

**Table 3:** Concomitant variables as predictors of class/component probabilities; coefficients represent associations with Class 2 relative to Class 1.

	Coef.	exp(Coef.)	SE	z	*p*	Lower 95%	Upper 95%
**log_hs**	0.070	1.073	0.018	3.860	<0.001	0.035	0.106
**herd_type_num**							
**Beef**	<0.001	1.000					
**Dairy**	0.158	1.171	0.061	2.590	0.010	0.039	0.277
**Other**	−0.331	0.718	0.099	−3.350	0.001	−0.525	−0.137
**Suckler**	−0.096	0.909	0.051	−1.870	0.061	−0.196	0.005

**Table 4:** Estimated associations between factors and breakdown duration (log_length) for Class 1 and Class 2 subpopulations from a finite mixture model.

Log_Length	Class 1 “Central”				Class 2 “Tail”			
	Coef.	*p*	Lower 95%	Upper 95%	Coef.	*p*	Lower 95%	Upper 95%
**log_hs**	<0.001	0.835	−0.003	0.002	0.090	<0.001	0.077	0.102
**herd_history**								
**TB free**	**ref**				**ref**			
**Zero reactors**	−0.001	0.787	−0.009	0.007	0.115	<0.001	0.068	0.162
**1 reactors**	0.015	<0.001	0.007	0.023	0.129	<0.001	0.076	0.182
**2–4 reactors**	0.017	<0.001	0.009	0.025	0.219	<0.001	0.166	0.273
**≥5 reactors**	0.017	0.004	0.005	0.029	0.226	<0.001	0.155	0.296
**herd_type_num**								
**Beef**	**ref**				**ref**			
**Dairy**	0.025	<0.001	0.018	0.031	−0.075	<0.001	−0.114	−0.036
**Other**	0.003	0.560	−0.006	0.012	−0.095	0.003	−0.157	−0.033
**Suckler**	0.006	0.015	0.001	0.011	−0.014	0.373	−0.046	0.017
**Controlled finishing units**								
**Never**	**ref**				**ref**			
**Designated**	−0.001	0.932	−0.030	0.027	0.710	<0.001	0.603	0.818
**log_NPH**	−0.003	0.040	−0.005	<0.001	−0.014	0.054	−0.028	<0.001
**parcel_cut**								
**1**	**ref**				**ref**			
**2**	<0.001	0.902	−0.006	0.006	0.026	0.177	−0.012	0.063
**3**	0.004	0.224	−0.002	0.010	0.031	0.110	−0.007	0.069
**4**	0.005	0.116	−0.001	0.012	0.064	0.002	0.023	0.105
**≥5**	0.001	0.736	−0.005	0.007	0.049	0.006	0.014	0.084
**med_area**	<0.001	<0.001	<0.001	<0.001	<0.001	0.299	<0.001	0.001
**cut_mmoves**								
**<90**	**ref**				**ref**			
**>90**	−0.039	<0.001	−0.047	−0.032	0.108	<0.001	0.062	0.155
**bd_start_yr**	<0.001	0.470	−0.001	0.001	−0.023	<0.001	−0.029	−0.017

**Table 5:** Summary statistics of outcome and covariate parameters for the FMM model of bTB breakdown duration.

Variable	n	Mean	Std. Dev.	exp(Mean)	Min	Max
**log_length**	17,312	5.087	0.403	161.869	0.095	7.736
**log_hs**	17,312	4.058	1.162	57.887	<0.001	7.385
**log_NPH**	17,312	4.397	0.986	81.210	−4.605	6.733
**med_area**	17,312	49.360	39.962		<1.000	754.900
**herd_history**						
**TB free**	13,833	80%			0	1
**Zero reactors**	1156	7%			0	1
**1 reactor**	896	5%			0	1
**2–4 reactors**	935	5%			0	1
**≥5 reactors**	492	3%			0	1
**herd_type**						
**Beef**	3576	21%			0	1
**Dairy**	4743	27%			0	1
**Other**	830	5%				
**Suckler**	8163	47%			0	1
**Feedlot**						
**0**	17,173	99%				
**1**	139	1%			0	1
**parcel_cut**						
**1**	3030	18%				
**2**	3299	19%			0	1
**3**	3069	18%			0	1
**4**	2425	14%			0	1
**≥5**	5489	32%			0	1
**cut_mmoves**						
**0**	15,660	90%				
**1**	1652	10%			0	1
**bd_start_yr**						
**2012**	1231	7%				
**2013**	2473	14%			0	1
**2014**	2466	14%			0	1
**2015**	2486	14%			0	1
**2016**	2571	15%			0	1
**2017**	2935	17%			0	1
**2018**	3150	18%			0	1

## Supplementary Materials

The following are available online at https://www.mdpi.com/2076-0817/9/10/815/s1, Figure S1: Output plots of the class probability and predicted log(breakdown duration) from an FMM of bTB breakdowns from a full model, Figure S2: Relationship between harm area (Ha) or herd size (number of cattle) and total bovine organic nitrogen per farm (kg). Figure S3: Relationship between predicted duration and log(NPH) for non-feedlot herds from an FMM model excluding log-herd size and farm area (ha). Figure S4: Schematic of model construction steps. Table S1: FMM log-normal parameter estimates for a model excluding log-herd size and farm area (ha) with two-components/classes. Class probabilities were modelled with variation across herd types.
