# Supplementary material for: Bovine Tuberculosis (Mycobacterium bovis) Outbreak Duration in Cattle Herds in Ireland: A Retrospective Observational Study
Source: Pathogens. 2020 Oct 5;9(10):815. doi: 10.3390/pathogens9100815 (PMC7650827; doi:10.3390/pathogens9100815)
Supplement: Supplementary file 1 [file pathogens-09-00815-s001.zip › supplementary/Table S1.docx]

**Table 1.** FMM log-normal parameter estimates for a model excluding log-herd size and farm area (ha) with two-components/classes. Class probabilities were modelled with variation across herd types.

| Outcome: log length of breakdown | | |  |  |  |  |  |  |
| --- | --- | --- | --- | --- | --- | --- | --- | --- |
|  | **Class 1 "Central"** |  |  |  | **Class 2 "Tail"** |  |  |  |
|  | **Beta** | **p** | **Lower 95%** | **Upper 95%** | **Beta** | **p** | **Lower 95%** | **Upper 95%** |
| herd_history |  |  |  |  |  |  |  |  |
| TB free | ref |  |  |  |  |  |  |  |
| Zero reactors | -0.002 | 0.624 | -0.010 | 0.006 | 0.135 | 0.000 | 0.088 | 0.183 |
| 1 reactors | 0.014 | 0.001 | 0.006 | 0.022 | 0.151 | 0.000 | 0.098 | 0.205 |
| 2-4 reactors | 0.016 | 0.000 | 0.008 | 0.024 | 0.250 | 0.000 | 0.196 | 0.304 |
| >=5 reactors | 0.014 | 0.015 | 0.003 | 0.026 | 0.267 | 0.000 | 0.195 | 0.339 |
|  |  |  |  |  |  |  |  |  |
| herd_type_num |  |  |  |  |  |  |  |  |
| Beef | ref |  |  |  |  |  |  |  |
| Dairy | 0.019 | 0.000 | 0.013 | 0.026 | 0.020 | 0.306 | -0.018 | 0.057 |
| Other | 0.002 | 0.722 | -0.007 | 0.011 | -0.077 | 0.015 | -0.139 | -0.015 |
| Suckler | 0.005 | 0.040 | 0.000 | 0.010 | 0.002 | 0.909 | -0.030 | 0.034 |
| Feedlot |  |  |  |  |  |  |  |  |
| Never | ref |  |  |  |  |  |  |  |
| Designated | -0.001 | 0.939 | -0.030 | 0.028 | 0.737 | 0.000 | 0.628 | 0.846 |
| log_NPH | -0.002 | 0.092 | -0.004 | 0.000 | 0.012 | 0.076 | -0.001 | 0.025 |
| parcel_cut |  |  |  |  |  |  |  |  |
| 1 | ref |  |  |  |  |  |  |  |
| 2 | -0.001 | 0.855 | -0.007 | 0.006 | 0.036 | 0.060 | -0.002 | 0.074 |
| 3 | 0.003 | 0.313 | -0.003 | 0.010 | 0.051 | 0.009 | 0.012 | 0.089 |
| 4 | 0.004 | 0.210 | -0.002 | 0.011 | 0.091 | 0.000 | 0.050 | 0.132 |
| >=5 | -0.002 | 0.577 | -0.007 | 0.004 | 0.095 | 0.000 | 0.061 | 0.129 |
| cut_mmoves |  |  |  |  |  |  |  |  |
| <90 |  |  |  |  |  |  |  |  |
| >90 | -0.045 | 0.000 | -0.052 | -0.038 | 0.178 | 0.000 | 0.133 | 0.223 |
| bd_start_yr | 0.000 | 0.637 | -0.001 | 0.001 | -0.022 | 0.000 | -0.028 | -0.016 |
| _cons | 4.480 | 0.000 | 2.484 | 6.476 | 49.086 | 0.000 | 37.088 | 61.083 |
