# Supplementary figures and images for: Bovine Tuberculosis (Mycobacterium bovis) Outbreak Duration in Cattle Herds in Ireland: A Retrospective Observational Study
Source: Pathogens. 2020 Oct 5;9(10):815. doi: 10.3390/pathogens9100815 (PMC7650827; doi:10.3390/pathogens9100815)

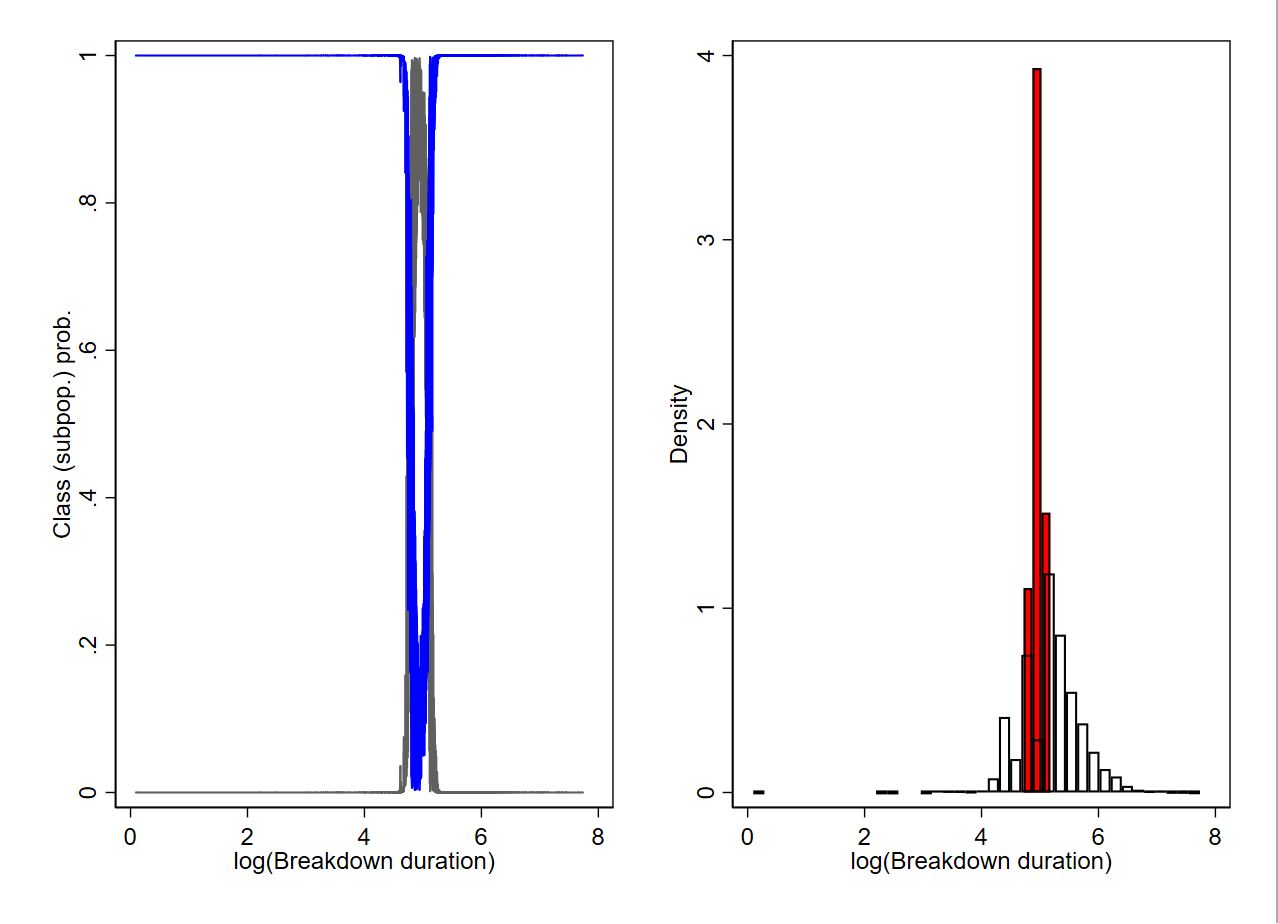

Supplement: Supplementary file 1 [file pathogens-09-00815-s001.zip › supplementary/Figure S1.tif]

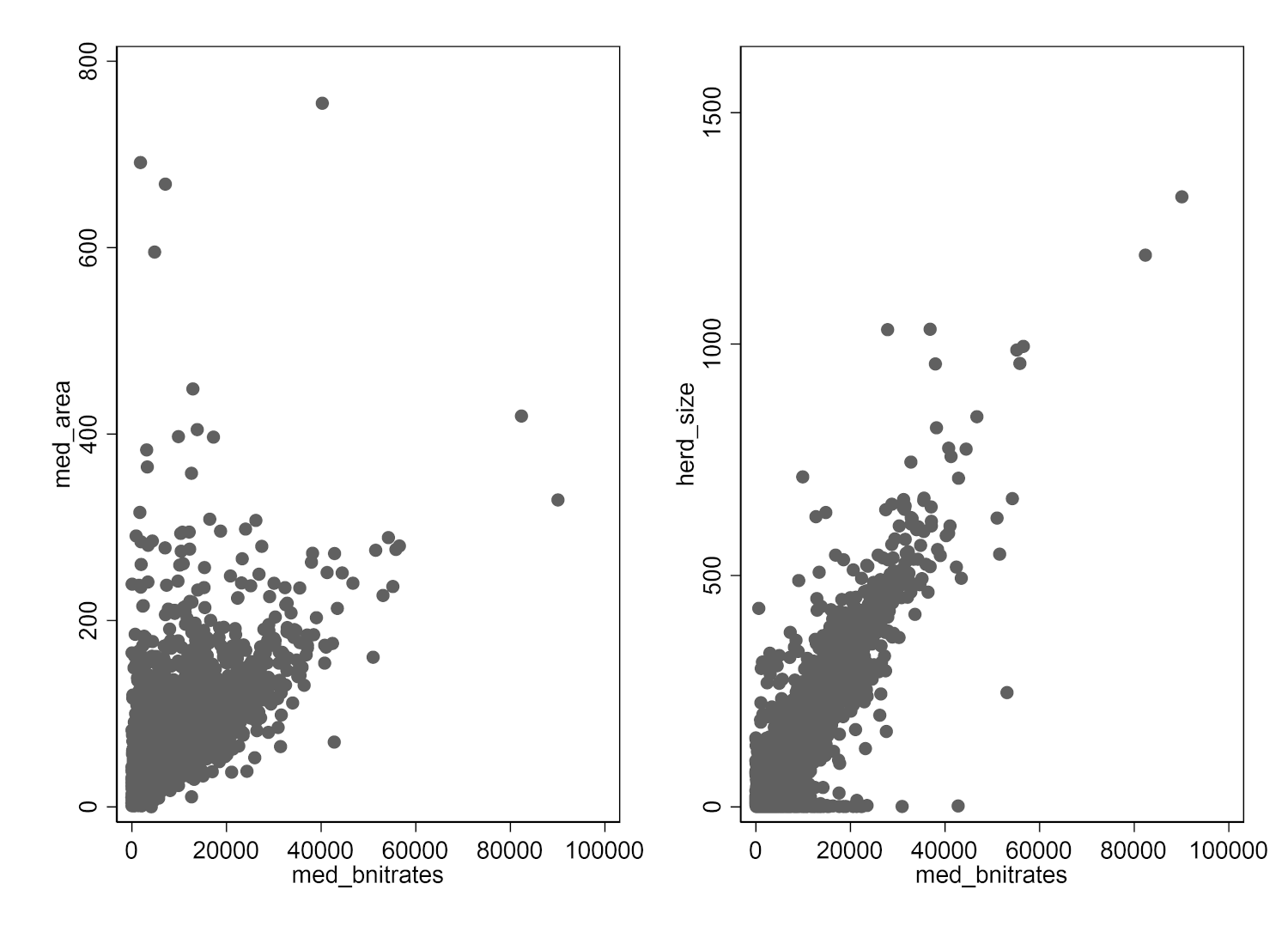

Supplement: Supplementary file 1 [file pathogens-09-00815-s001.zip › supplementary/Figure S2.tif]

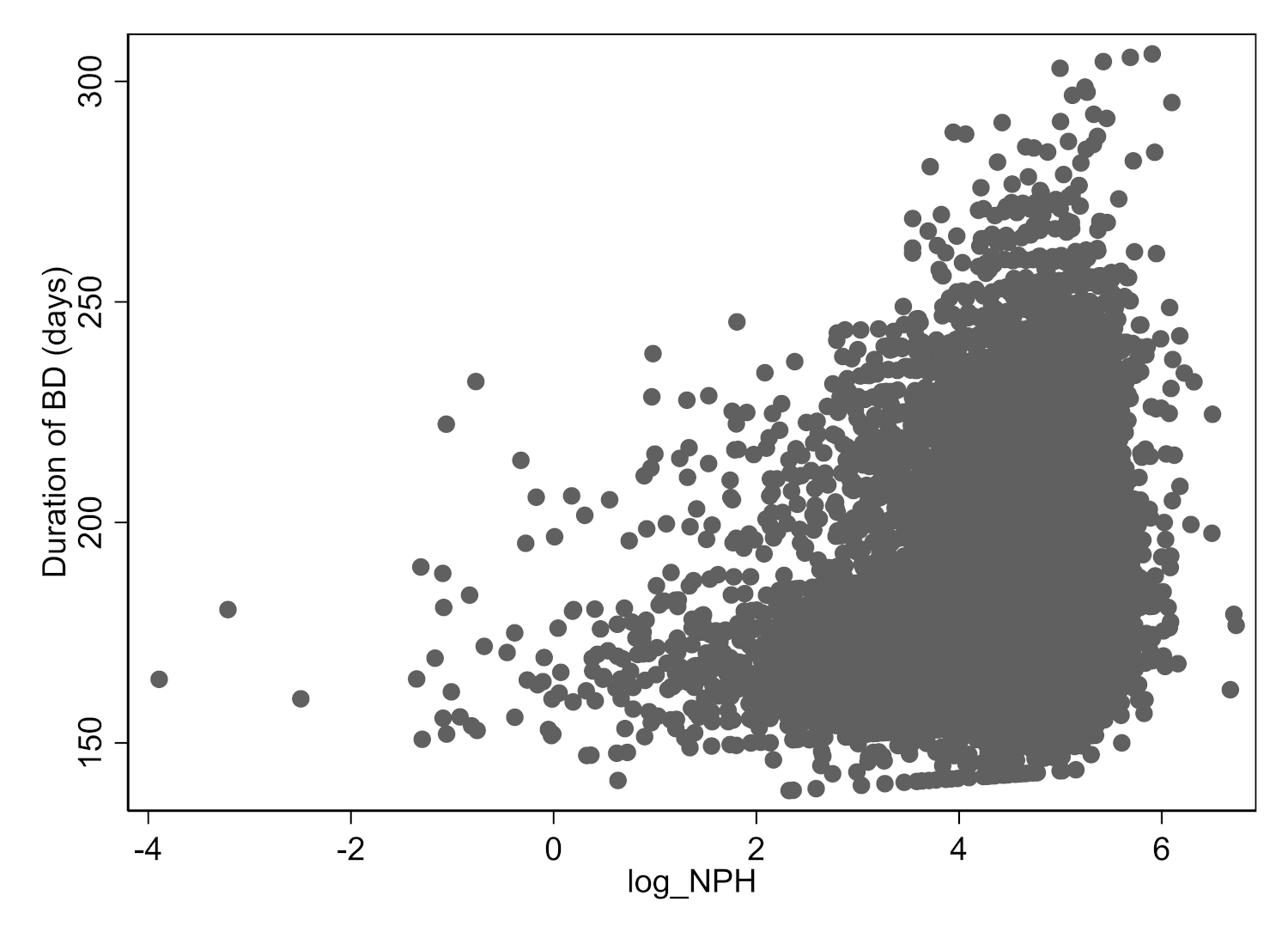

Supplement: Supplementary file 1 [file pathogens-09-00815-s001.zip › supplementary/Figure S3.tif]

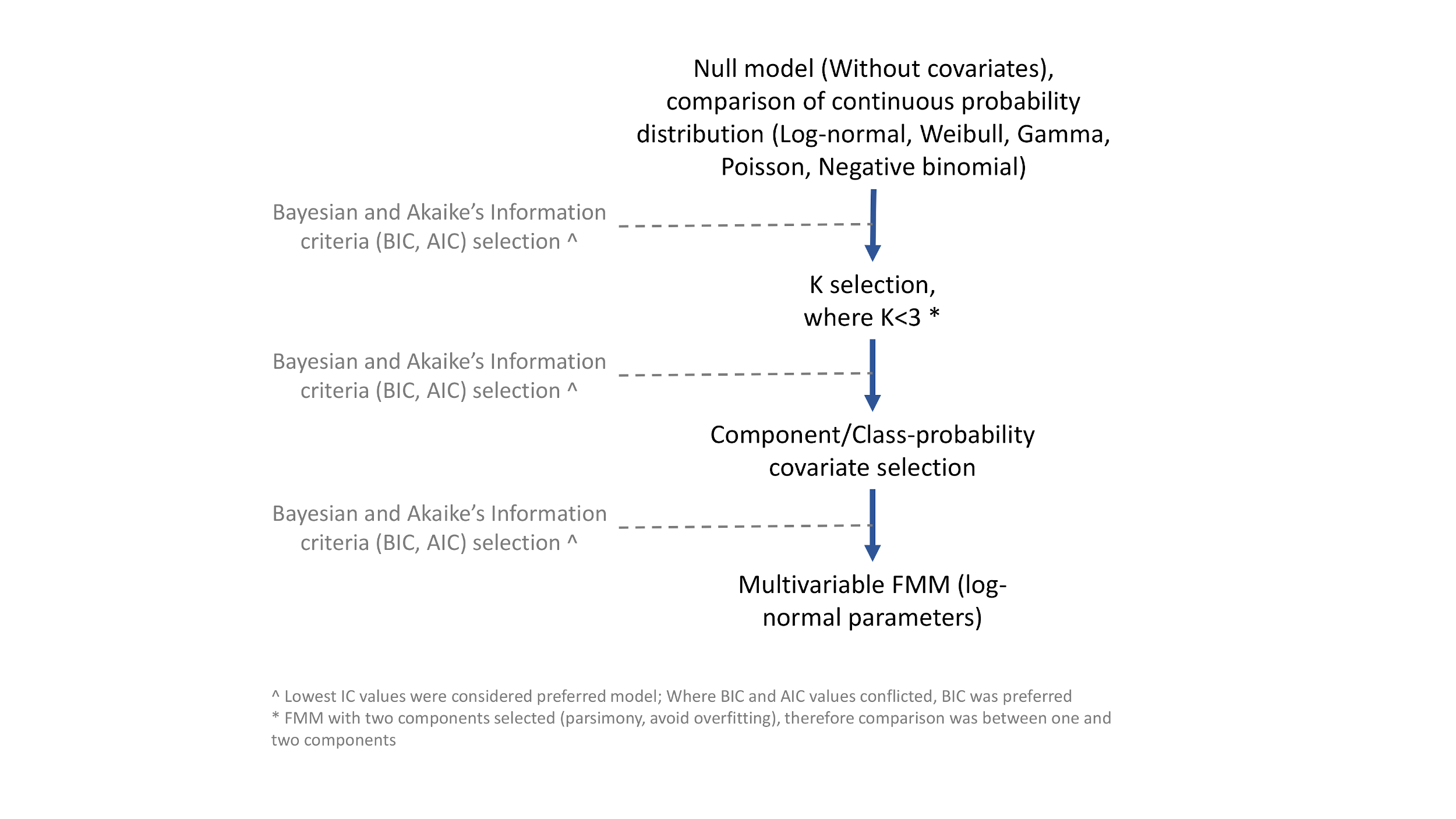

Supplement: Supplementary file 1 [file pathogens-09-00815-s001.zip › supplementary/Figure S4.tif]
